# Small-quantity lipid-based nutrient supplements containing different amounts of zinc along with diarrhea and malaria treatment increase iron and vitamin A status and reduce anemia prevalence, but do not affect zinc status in young Burkinabe children: a cluster-randomized trial

**DOI:** 10.1186/s12887-016-0765-9

**Published:** 2017-02-02

**Authors:** Souheila Abbeddou, Elizabeth Yakes Jimenez, Jérome W. Somé, Jean Bosco Ouédraogo, Kenneth H. Brown., Sonja Y. Hess

**Affiliations:** 1 Department of Nutrition, Program in International and Community Nutrition, University of California, One Shields Avenue, Davis, CA 95616 USA; 2 Center for Education Policy Research, University of New Mexico, Albuquerque, NM USA; 3 Pacific Institute for Research and Evaluation, Albuquerque, NM USA; 4 Institut de Recherche en Sciences de la Santé, Bobo-Dioulasso, Burkina Faso; 5 Nutrition and Global Development, Bill & Melinda Gates Foundation, Seattle, WA USA

**Keywords:** Zinc, SQ-LNS, Lipid-based nutrient supplements, Hemoglobin, Anemia, Plasma zinc concentration, Iron, Vitamin A, Retinol-binding protein

## Abstract

**Background:**

We assessed the effects of providing a package of interventions including small-quantity lipid-based nutrient supplements (SQ-LNS) containing 0, 5 or 10 mg zinc and illness treatment to Burkinabe children from 9 to 18 months of age, on biomarkers of zinc, iron and vitamin A status at 18 months and compared with a non-intervention cohort (NIC).

**Methods:**

Using a two-stage cluster randomized trial design, communities were randomly assigned to the intervention cohort (IC) or NIC, and extended family compounds within the IC were randomly assigned to different treatment groups. IC children (*n* = 2435) were provided with 20 g SQ-LNS/d containing 0, 5 or 10 mg zinc, 6 mg of iron and 400 μg of vitamin A along with malaria and diarrhea treatment. NIC children (*n* = 785) did not receive the intervention package. At 9 and 18 months, hemoglobin (Hb), zinc, iron and vitamin A status were assessed in a sub-group (*n* = 404). Plasma concentrations of zinc (pZC), ferritin (pF), soluble transferrin receptor (sTfR) and retinol-binding protein (RBP) were adjusted for inflammation.

**Results:**

At baseline, 35% of children had low adjusted pZC (<65 μg/dL), 93% were anemic (Hb <110 g/L), 25% had low adjusted pF (<12 μg/L), 90% had high adjusted sTfR (>8.3 mg/L) and 47% had low adjusted RBP (<0.94 μmol/L), with no group-wise differences. Compared with the NIC, at 18 months IC children had significantly lower anemia prevalence (74 vs. 92%, *p* = 0.001) and lower iron deficiency prevalence (13% vs. 32% low adjusted pF and 41% vs. 71% high adjusted sTfR, *p* < 0.001), but no difference in pZC. Mean adjusted RBP was greater at 18 months in IC vs. NIC (0.94 μmol/L vs. 0.86 μmol/L, *p* = 0.015), but the prevalence of low RBP remained high in both cohorts. Within the IC, different amounts of zinc had no effect on the prevalence of low pZC or indicators of vitamin A deficiency, whereas children who received SQ-LNS with 10 mg zinc had a significantly lower mean pF at 18 months compared to children who received SQ-LNS with 5 mg zinc (*p* = 0.034).

**Conclusions:**

SQ-LNS regardless of zinc amount and source provided along with illness treatment improved indicators of iron and vitamin A status, but not pZC.

**Trial registration:**

NCT00944281 (July 21, 2009).

## Background

Zinc, iron and vitamin A are essential for optimal physical growth, cognitive development and immune function of young children [1–5]. Deficiencies of these micronutrients are prevalent in sub-Saharan Africa, where approximately one fourth of the population are at risk of zinc deficiency [6], ~20% of pre-school children suffer from iron deficiency anemia [7], and >40% of children have subclinical vitamin A deficiency, based on serum retinol concentration <0.70 μmol/L [8]. These micronutrient deficiencies often co-exist in low-income populations, and combined supplementation with zinc, iron and vitamin A is gaining traction as a strategy to decrease childhood morbidity and mortality [3, 5, 9, 10].

There are multiple potential interactions among micronutrients when they are co-supplemented. For example, some studies found that adding zinc to iron supplements reduced their impact on iron status, although a meta-analysis of available studies concluded that there was no significant effect of concomitant zinc supplementation on the response of iron status indicators and hemoglobin (Hb) to iron supplementation [10, 11]. Zinc may also affect vitamin A metabolism through its involvement in protein synthesis and cellular enzyme functions [12], but there are only a limited number of studies that have evaluated the effect of combined zinc and vitamin A supplementation on vitamin A status, and the results are inconclusive [12–14].

Home fortification of children’s foods using small-quantity lipid-based nutrient supplements (SQ-LNS) providing ~110–120 kcal/day (20 g dose) or medium-quantity lipid-based nutrient supplements (MQ-LNS) providing ~250–500 kcal/day (45–90 g dose) is a promising strategy to support normal linear growth and development in young children [15]. Despite reports of beneficial effects of SQ- and MQ-LNS on children’s growth [16–18], there is little research on their effects on micronutrient status, and the available studies have assessed only a few micronutrients. Plasma zinc concentration (pZC) did not differ between Malawian children who received 4.5–7.0 mg zinc via either MQ-LNS (40–60 g) or isocaloric fortified porridge from 6 to 18 months of age [18]. Daily supplementation of 6–12 months old Ghanaian children with SQ-LNS reduced iron deficiency anemia by 32%, but did not affect zinc status [19]. A recent study from Honduras found that supplementing children with MQ-LNS (46 to 70 g; containing 9 mg zinc, 9 mg iron, 400 μg vitamin A and 0.9 μg vitamin B12 per 46 g) from age 6 to 18 months had no effect on Hb, iron or zinc status, but significantly reduced vitamin A and vitamin B_12_ deficiencies [20].

The objectives of the present study were to assess 1) the effects of supplementing Burkinabe children from 9 to 18 months of age with different amounts of zinc (0, 5 or 10 mg zinc) in SQ-LNS containing 6 mg iron and 400 μg vitamin A along with the treatment of diarrhea and malaria on biomarkers of zinc, iron and vitamin A status at 18 months; and 2) the combined effect of SQ-LNS and diarrhea and malaria treatments on these indicators compared with a non-intervention cohort (NIC).

## Methods

### Study design

This study was a cluster randomized, partially double-masked, placebo-controlled trial (the International Lipid-based Nutrient Supplement-Zinc Trial, iLiNS-ZINC), which took place in the Dandé Health District in southwestern Burkina Faso from April 2010 to July 2012. Ethical approval was provided by the Institutional Review Boards of the Centre Muraz in Bobo-Dioulasso (Burkina Faso) and the University of California, Davis (USA). Caregivers provided separate written, informed consents for participation in the study and for collection of biological specimens from the child. The study was registered as a clinical trial with the U.S. National Institute of Health (https://www.clinicaltrials.gov/ct2/results?term=NCT00944281).

**Fig. 1:**
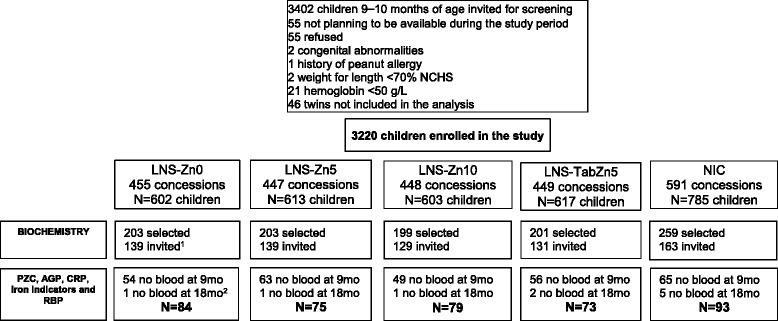
Flow diagram of the biochemistry indicators for iLiNS-Zinc. ^1^In all groups, invited if free of confirmed fever or diarrhea during the enrollment day. ^2^In all groups, no sample at 18 mo either because child failed to provide biological samples or because of dropout

This trial included two levels of randomization: 1) the community and 2) the concession (extended family compound). Thirty-four communities accessible during the rainy season were stratified by health clinic catchment area and randomly allocated to intervention cohort (IC, 25 communities) or non-intervention cohort (NIC, 9 communities), in such a way to ensure balanced cohorts with respect to population size, distance from a paved road, and distance from the city of Bobo-Dioulasso. A total of 3220 children 9–10 month of age (~95% of those screened) were enrolled in the study after meeting all the enrollment criteria (Fig. 1). Of these, 2435 children were included in the IC and 785 in the NIC. Children were considered eligible if they were 8.8 to 9.9 months of age, were permanent residents of the study area, and their caregivers planned to be available during the study period and accepted weekly home visits. Children were not enrolled in the study when their Hb was <50 g/L, weight-for-length was <70th percentile of the National Center for Health Statistics/World Health Organization (NCHS/WHO) growth reference [21], or they had any illness warranting hospital referral or potentially interfering with growth, as reported previously in greater detail [22, 23] (Fig. 1).

### Randomization and blinding

Within the intervention cohort, individual concessions were randomly assigned to one of eight color codes using a block randomization list prepared by a statistician from the University of California Davis. Two colors represented each treatment group, and all participants, field staff, study statistician and investigators were blinded to the intervention groups during the trial. Cluster randomization at the level of the concession was chosen to reduce the risk of cross-contamination within the family compound through food sharing.

### Intervention

Children in the IC were assigned to receive one of the following daily supplements from 9 to 18 months of age: 1) SQ-LNS without added zinc, and placebo tablet (LNS-Zn0); 2) SQ-LNS with 5 mg added zinc, and placebo tablet (LNS-Zn5); 3) SQ-LNS with 10 mg added zinc, and placebo tablet (LNS-Zn10); or 4) SQ-LNS without added zinc, and 5 mg zinc tablet (LNS-TabZn5). Supplementation of children in IC started the day after the baseline screening. Children in the NIC (*N* = 785) did not receive SQ-LNS or tablets from 9 to 18 months of age, but received SQ-LNS with 10 mg zinc for 9 months beginning at 18 mo of age after the final blood sample was collected. Caregivers in the IC communities were instructed to administer 20 g SQ-LNS per day in two separate servings, preferably mixed in a small portion of the child’s meal, and to give the dispersible tablet once daily at least 30 min away from meals. At enrollment and throughout the study, the caregivers were advised to continue breastfeeding and feed diverse foods to the child, and IC caregivers were reminded that SQ-LNS should not replace other components of the diet. Adherence was assessed by obtaining reported information on SQ-LNS and tablet consumption, collecting any remaining SQ-LNS and tablets and empty packages, and by direct observation in a subgroup of children, as reported in more detail elsewhere [24].

At baseline, all children were treated for anemia, fever, malaria, and caregiver reported diarrhea following the national health policy in Burkina Faso. Children with Hb <80 g/L received iron supplements (ferrous sulfate, 2–6 mg iron/kg body weight/d for 30 days, depending on the anemia severity) and 200 mg mebendazole/d for three days to treat possible helminthic infections. Children with a positive rapid diagnostic test (RDT) for malaria received malaria treatment for three days (amodiaquine-artesunate, 1 tablet/d) and an antipyretic (paracetamol, 1/2 tablet/d for three days), and children with confirmed fever and a negative RDT and no other clinical symptoms received paracetamol only for three days. Oral rehydration salts (ORS: 1 sachet/d for 4 days) were given to children with reported diarrhea. Caregivers in the IC were advised to start the supplementation even during illness.

### Biochemistry subgroup

A subset of 1065 children from the IC and NIC were randomly selected for the biochemistry subgroup and asked to provide a venous blood sample as described below. The biochemistry subgroup included only one child from a concession to avoid reduced accuracy of estimation due to intra-cluster correlation, and excluded children with reported fever or diarrhea symptoms on the day of enrollment or on the scheduled blood collection day. Children were enrolled in the biochemistry subgroup until the target sample size was reached. This paper reports on data obtained from children who successfully provided blood specimens at both 9 and 18 months for analyses of micronutrient status.

### Sample size for the biochemistry subgroup

The sample size estimate for the micronutrient status assessments was based on the number of children needed in each group to detect differences in effect size of 0.6 SDs with a significance of *p* ≤ 0.05 and power ≥0.80 for group-wise comparisons of pZC among the means of five groups (the four intervention groups and the NIC), consistent with the magnitude of effect reported in previous zinc supplementation trials [10, 25]. The estimated sample size for the NIC was inflated for an assumed design effect of 1.5 due to the cluster sampling design, resulting in an estimated total sample requirement of 374 children in the 5 groups (68 in each of the 4 intervention groups and 102 in the NIC). This target sample size was increased to a total of 468 children in the 5 groups to allow for 20% attrition.

### Anthropometry

When the children were 9 months of age, their length was measured to the nearest 0.1 cm (Seca Model 417, Hamburg, Germany), and their weight was assessed with 50 g precision (Seca Model 383). The mothers’ height (Seca Model 217) and weight (Seca Model 874) were assessed during the same visit. Measurements were carried out in duplicate by one of four trained and standardized anthropometrists and their assistants. A third measurement was carried out in case of a disagreement between the two first measurements of >0.5 cm for length/height and >0.1 kg for weight. The average of the two closest values was used in the statistical analysis.

### Household demographics and child feeding and morbidity data

Data on maternal education and marital status were collected at enrollment. At 9 and 18 months, 24-h and 7-day recall data on food intake frequencies and breastfeeding patterns were collected for all children. After enrollment, children in the IC were visited weekly by trained field agents who delivered the supplements and collected data on adherence to supplementation [24], general health status and morbidity symptoms [23]. Treatment was provided in case of reported diarrhea, reported or confirmed fever, and confirmed malaria, as described above. Data on high-dose vitamin A supplementation was collected by the field agents on a monthly basis. The field agent showed a picture of a high-dose vitamin A capsule during the interview to help the mother recognize it and distinguish it from the oral polio vaccine.

### Capillary blood hemoglobin and zinc protoporphyrin concentrations

At the time of enrollment screening, Hb was measured in a capillary blood specimen, using a Hemocue device (model Hb 201+, Ängelholm, Sweden), and 300 μL capillary blood was collected in microcuvettes containing lithium heparin (CB 300 LH, Sarstedt AG & Co, Nümbrecht, Germany) and stored in a cold-box for subsequent analysis of zinc protoporphyrin (ZPP) [26], as described below.

### Venous blood collection and processing

Trained phlebotomists also obtained 5 mL blood from an antecubital or metacarpal vein from children in the biochemistry subgroup. The venous specimen was collected into trace element-free lithium heparin vacutainer tubes (Sarstedt AG & Co, Nümbrecht, Germany) 1–2 h after the last breastfeeding episode, using the specimen collection and processing methods recommended by the International Zinc Nutrition Consultative Group [4]. Blood samples were stored in a cold-box and transported to the field laboratory for processing and/or analysis. At the field laboratory, plasma was separated from whole blood by centrifuging at 2800 rpm for 10 min. For analysis of pZC, iron status indicators, retinol-binding protein (RBP) and acute phase proteins [C-reactive protein (CRP) and α-1-acid glycoprotein (AGP)], plasma was stored at −20 °C until shipment to the collaborating laboratories for analysis.

### Laboratory analyses

PZC was measured using an inductively coupled plasma optical emission spectrophotometer (Vista; Varian Inc, Walnut Creek, CA) at the Children’s Hospital of Oakland Research Institute [27, 28]. Inter- and intra-run coefficients of variation (CV) of control serum were 2.3% and 1.7%, respectively. Indicators of iron status [plasma ferritin (pF), soluble transferrin receptor (sTfR)] and vitamin A status [RBP], and acute phase proteins [CRP and AGP] were analyzed by ELISA (DBS-Tech in Willstaett, Germany) [29]. The CVs of the different indicators for a pooled plasma sample were 3.3%, 2.0%, 3.3%, 4.4% and 5.5% for pF, sTfR, RBP, CRP and AGP, respectively. Retinol (ROH) concentration was measured in a subset of randomly selected samples by using high performance liquid chromatography (HPLC, Agilent 1100) at the University of California, Davis, using a slight modification of the method described by Bieri et al. [30]. The CV of a control sample run in triplicate with each batch of plasma ROH samples was 2.6% within the same run and 2.7% between runs. ZPP was assessed in capillary blood samples at the field laboratory using a hematofluorometer (206D, AVIV Biomedical Inc., Lakewood, NJ, USA) after a maximum of 4 days of storage in the refrigerator, during which ZPP is considered stable [31]. Samples were analyzed in duplicate, unless inadequate volume only allowed for one measurement.

### Validation of the RBP cutoff for vitamin A deficiency

Cutoffs for determining vitamin A deficiency based on RBP differ among populations [32], so we determined a cutoff for our study population by measuring both ROH and RBP concentrations in a subset of 40 randomly selected samples. Logarithm-transformed ROH values were tested for their correlation with logarithm-transformed RBP values from the same child at the same age using the Pearson correlation test, and a linear regression was generated (Proc GLM). There was a high correlation between ROH and RBP measured in the 40 samples (*R* = 0.78, *p* < 0.0001). To derive the corresponding RBP cutoff for vitamin A deficiency, a regression model used was: Log ROH = −0.30 + 0.87 (log RBP). In this population, the RBP cutoff corresponding to 0.70 μmol ROH/L is 0.94 μmol RBP/L. The sensitivity of this population-adjusted cutoff to detect low ROH (<0.70 μmol/L) was 82% and the specificity was 83%.

### Data analysis

All statistical analyses were carried out using SAS software for Windows (9.3, SAS Institute, Cary, North Carolina). Descriptive statistics (means, geometric means (95% confidence interval) and proportions) were used to assess baseline information by group, and to compare children included in the biochemistry subgroup with those not included. Variables that were not normally distributed were transformed using Box-Cox transformations to find the optimal transformation. Analysis was done with logarithm-transformed variables, which were back transformed for reporting of descriptive results.

Plasma concentrations of zinc, pF, sTfR and RBP and capillary whole blood ZPP were adjusted categorically for the presence of inflammation [33]. Participants were stratified into four inflammation categories based on elevation of one or both acute phase proteins, or no inflammation using the method described by Thurnham et al. [33], and cutoffs of 5 mg/L for CRP and 1 g/L for AGP. Body iron stores (BIS) were calculated using the ratio of both adjusted and unadjusted sTfR and pF concentrations using the formula developed by Cook et al. [34]. Micronutrient deficiencies were defined using the following cutoffs, for both adjusted and unadjusted values: zinc deficiency (pZC <65 μg/dL) [4], anemia (Hb <110 g/L) [35], iron deficiency [ID, pF <12 μg/L [36], sTfR >8.3 mg/L, BIS <0 mg/kg, and ZPP >70 μmol/mol heme [37]], and vitamin A deficiency (RBP <0.94 μmol/L). Additionally, iron deficiency anemia (IDA) was defined as having both anemia and ID, as outlined above.

Length-for-age z-score (LAZ) and weight-for-length z-score (WLZ) were calculated in relation to the World Health Organization Child Growth Standards using SAS macros [38]. Breastfeeding and 24 h-complementary feeding indicators were constructed according to World Health Organization guidelines [39]. 7-day intake of vitamin-A rich fruits and vegetables were summarized as continuous scores ranging from 0 to 7. Consumption frequencies of vitamin A-rich fruits and vegetables were also categorized dichotomously (<3 or ≥3 d/week). Definitions of infectious diseases identified in IC children are reported in more detail elsewhere [22, 23]. Briefly, diarrhea was defined as caregiver report of three or more liquid or semi-liquid stools during a 24-h period. An episode of diarrhea was defined as the period starting the day the child first had diarrhea preceded and followed by a two-day, diarrhea-free period. Fever was defined as any fever reported by the caregiver or elevated auricular temperature (>37.5 °C), as measured by the field workers. An episode of fever was defined as the period starting the day the child first had fever preceded and followed by two days when the child had not had fever. Malaria was defined as the presence of reported or confirmed fever during the 24 h preceding the morbidity visit, associated with a positive RDT. An episode of malaria was defined as the presence of a new episode of reported or confirmed fever and a positive malaria RDT obtained >21 days after a previous treated malaria episode.

Outcomes for inflammation, zinc, iron and vitamin A status and changes in the outcomes between baseline and at 18 months were compared by intervention group and cohort using analysis of covariance for continuous outcome variables and logistic regression for dichotomous variables. Differences in the outcomes between baseline and at 18 months were tested using repeated measurement of covariance for continuous outcomes and the random statement of the logistic regression for dichotomous variables. Analysis included a random effect of the community to account for intra-community correlation. Intervention group and cohort were used as the main effects, and covariates were tested individually in a binary model with the outcome before including the variables with *p* < 0.1 in the final model. All the outcomes were adjusted for baseline values. Covariates tested were previously listed in our plan of analysis, which was written before the study code was broken, and included child age, sex, baseline anthropometric characteristics and maternal and household characteristics, study season and 18 months acute phase proteins. Indicators of iron status and RBP were adjusted additionally for baseline RDT, iron supplementation and breastfeeding and 24-h complementary feeding indicators. Covariates which were significantly associated with the outcome were tested for their potential modifying effect of the intervention groups and cohort. We also tested baseline pZC for its potential modifying effect of the intervention cohort and group, and recent high-dose vitamin A supplementation of the intervention groups on RBP at 18 months within the IC. Intervention group means were compared post-hoc using least-square means with the Tukey-Kramer test. Tables report the comparison among the four intervention groups and between the combined intervention groups and the non-intervention cohort (IC vs. NIC). Additional 5-group comparisons were conducted and reported only when deemed helpful to clarify subtle differences in the results.

## Results

### Baseline characteristics

Among the 1065 children randomly selected for possible participation in the biochemistry sub-group, 66% (*n* = 701) were eligible on the scheduled day (i.e. free of symptomatic diarrhea and fever, as reported by the caregiver). Among the eligible children, a venous blood sample was successfully collected in 60% (*n* = 414) at 9 months. Among those, 404 children successfully provided blood samples at both 9 and at 18 months and are included in this analysis (Fig. 1).

Children who provided venous blood samples did not differ from those who did not, except that a higher proportion of the children who provided samples had a positive initial RDT (67 vs. 61% respectively, *p* = 0.015). At baseline, 23% of children in the biochemistry subgroup were stunted, 32% were underweight and 18% were wasted. Anemia prevalence was high, affecting 93% of children in the biochemistry subgroup. At enrollment, one-third of the children had Hb concentration <80 g/L and received iron supplementation and antihelminthic treatment, and two-thirds were treated for positive malaria RDT. All children were still breastfed, and almost all children had started eating some type of complementary food. In particular, 83% of children reportedly received cereal-based foods and only 7 and 13%, respectively, consumed legumes and flesh food on the previous day (Table 1).

**Table 1:** Child, maternal and household characteristics of study participants assessed for biochemical status, by study group

	LNS-Zn0	LNS-Zn5	LNS-Zn10	LNS-TabZn	NIC	*p*-value for the difference among 5 groups^a,b^
N (children with 9 and 18 mo values)	84	75	79	73	93	
N boys (%)	38 (45.2)	41 (54.7)	31 (39.2)	38 (52.0)	40 (49.5)	1.000
Baseline child LAZ	−1.29 ± 1.00	−1.25 ± 1.04	−1.14 ± 1.08	−1.11 ± 1.10	−1.14 ± 1.08	0.783
Baseline child WLZ	−1.03 ± 1.20	−0.92 ± 1.04	−1.13 ± 0.91	−0.86 ± 1.05	−1.05 ± 1.11	0.506
N Baseline iron supplementation (%)^c^	20 (23.8)	21 (28.0)	21 (26.6)	23 (31.5)	29 (31.2)	0.828
N Baseline RDT positive (%)	62 (73.8)	49 (65.3)	54 (68.3)	47 (64.4)	58 (62.4)	0.479
Maternal body mass index (kg/m^2^)	20.5 ± 1.7	20.8 ± 2.3	21.1 ± 2.6	21.0 ± 2.3	20.4 ± 2.3	0.164
Maternal education						0.383
No formal or informal education	53 (63.1)	47 (62.7)	51 (64.6)	43 (58.9)	68 (73.1)	
Any informal education and/or less than one year of formal education	24 (28.6)	21 (28.0)	19 (24.0)	18 (24.7)	17 (18.3)	
At least one year of formal education	7 (8.3)	7 (9.3)	9 (11.14)	12 (16.4)	8 (8.6)	
Baseline child feeding practices						
Child still breastfed (%)	84 (100)	75 (100)	79 (100)	73 (100)	93 (100)	1.000
Animal source food^d^	21 (25.0)	18 (24.0)	15 (19.0)	22 (30.1)	22 (23.7)	0.621
Child morbidity during the intervention^e^						
Child malaria incidence (episodes per 100 child days at risk)	0.60 ± 0.48	0.62 ± 0.53	0.58 ± 0.48	0.64 ± 0.54	–	0.339
Child fever incidence (episodes/100 child-days at risk)	1.28 ± 0.84	1.34 ± 0.94	1.51 ± .1.19	1.39 ± 0.95	–	0.683
Child diarrhea incidence (episodes per 100 child days at risk)	1.02 ± 0.90	0.82 ± 0.66	0.92 ± 0.80	0.92 ± 0.92	–	0.469

*LAZ* length for age z-score, *WLZ* weight for length z-score

^a^Values presented are means ± SD, *n* (%)

^b^ *P*-values are from mixed model for continuous variables, logistic regression for binary variables and Chi square for polychotomous variables. All analyses were adjusted for the random effect of village, and in morbidity outcomes, were controlled for over-dispersion

^c^Children with Hb <80 g/L received ferrous sulfate, 2–6 mg iron/kg body weight/d for 30 days, depending on the anemia severity and 200 mg mebendazole/d for three days to treat possible helminthic infections

^d^Child consumed at least one animal-source food during the previous 24 h

^e^Morbidity outcomes include children in the IC who provided data on ≥30 days

### Inflammation indicators

At baseline, 4% of children had elevated CRP only, 33% had both elevated CRP and AGP and 30% had elevated AGP only, with no significant differences among the intervention groups or cohorts. Mean AGP and CRP concentrations at 18 months did not differ significantly by intervention cohort or by intervention group within the IC. Compared to the NIC, the IC had a lower prevalence of children who had elevated CRP only and both elevated CRP and AGP, and a greater proportion of children who had elevated AGP only at 18 months (2, 27 and 31%, respectively for IC children compared to 5%, 37% and 23% for NIC children, *p* = 0.03), but the total percentage of children with inflammation remained high for both cohorts at 18 months.

### Plasma zinc concentration

At baseline, 35% of the children had low pZC after adjustment for inflammation. The proportion of children with low pZC increased from 9 to 18 months (*p* < 0.0001), but did not differ among the intervention groups or between the cohorts at 18 months (Table 2). From 9 to 18 months, mean pZC decreased significantly from 67.1 to 63.6 μg/dL in the NIC and from 69.5 to 64.8 μg/dL in the IC (*p* < 0.0001). The change in pZC was not significantly different between the two cohorts (*p* = 0.39), nor by intervention group (*p* = 0.84). AGP and CRP were significantly associated with pZC at 18 months, but did not modify the effect of intervention group or cohort on final pZC.

**Table 2:** Effects of SQ-LNS containing different amounts and sources of zinc provided along with malaria and diarrhea treatment on plasma zinc concentration and prevalence of low plasma zinc concentration in children at 9 and 18 months, by study group

	LNS-Zn0	LNS-Zn5	LNS-Zn10	LNS-TabZn5	*P*-value among 4 intervention groups^a,b^	IC	NIC	*P*-value between cohorts^a,b^	ICC^c^	DE^d^
Plasma zinc concentration (μg/dL)^e^										
N (9 and 18 mo)	84	74	79	73		310	93			
Unadjusted pZC at 9 months	66.2 (63.4, 69.0)	66.0 (63.5, 68.6)	68.9 (66.5, 71.3)	68.8 (65.8, 72.0)	0.255	67.4 (66.1, 68.8)	65.1 (62.5, 67.7)	0.078	0.00	1.0
Adjusted pZC at 9 months	68.0 (65.2, 70.9)	68.1 (65.6, 70.8)	71.0 (68.6, 73.5)	71.2 (68.1, 74.5)	0.212	69.5 (68.2, 70.9)	67.1 (64.5, 69.9)	0.090	0.00	1.0
Unadjusted pZC at 18 months	61.7 (59.2, 64.4)	62.5 (60.1, 65.0)	64.4 (61.9, 67.0)	64.5 (62.4, 66.6)	0.774	63.2 (62.0, 64.4)	61.6 (59.4, 63.8)	0.968	0.02	1.3
Adjusted pZC at 18 months	63.3 (60.7, 65.9)	64.2 (61.8, 66.7)	66.0 (63.5, 68.7)	65.9 (63.8, 68.1)	0.742	64.8 (63.6, 66.0)	63.6 (61.4, 65.8)	0.831	0.03	1.4
Change in adjusted pZC^f^	−4.9 (−6.93, −2.78)	−4.0 (−6.58, −1.41)	−4.8 (−7.46, −2.13)	−6.0 (−8.89, −3.05)	0.843	−4.9 (−6.16, −3.64)	−3.9 (−6.23, −1.51)	0.391	–	–
% Low plasma zinc concentration (pZC <65 μg/dL)^e^										
Low unadjusted pZC at 9 months, *N* (%)	38 (45.3)	34 (45.9)	28 (35.4)	33 (45.2)	0.498	133 (42.9)	44 (47.3)	0.399	–	–
Low adjusted pZC at 9 months, *N* (%)	29 (34.5)	28 (37.8)	22 (27.8)	27 (37.0)	0.587	106 (34.2)	36 (38.7)	0.370	–	–
Low unadjusted pZC at 18 months, *N* (%)	53 (63.1)	46 (62.2)	46 (58.2)	39 (53.4)	0.909	184 (59.3)	56 (60.2)	0.379	–	–
Low adjusted pZC at 18 months, *N* (%)	50 (59.5)	42 (56.8)	43 (54.4)	34 (46.6)	0.723	169 (54.5)	51 (54.8)	0.425	–	–

*pZC* plasma zinc concentration

^a^Geometric mean (95% confidence interval), *n* (%). Values in the same row with different superscript letters are significantly different (*P* < 0.05)

^b^Adjusting for the random effect of the village and baseline value

^c^Inter-cluster coefficient calculated based on the cluster (village) variance and the residual variance from proc MIXED

^d^Design effect calculated based on the standard error of the intervention with or without the random effect of the cluster

^e^pZC adjusted for time of blood draw, time since last breastfeed and CRP and AGP concentration; and additionally for baseline value at 18 months

^f^Means (95% CI) were calculated based on non-transformed adjusted values, but covariance analysis was done with the logarithmic-transformed variable

### Hemoglobin concentration

At baseline, the mean Hb concentration for all groups combined was 88 g/L, and 93% of the children were anemic. At 18 months, the prevalence of anemia decreased by one-fifth in the IC, but did not change in the NIC (Tables 3 and 4). Nevertheless, 74% of the children in the IC remained anemic, and there were no significant differences among the four intervention groups.

**Table 3:** Effect of SQ-LNS containing different amounts and sources of zinc provided along with malaria and diarrhea treatment on hemoglobin concentration and iron status indicators among children 9–18 months of age, by study group

	LNS-Zn0	LNS-Zn5	LNS-Zn10	LNS-TabZn5	*P*-value among 4 intervention groups^b^	IC	NIC	*P*-value between cohorts^b^	ICC^c^	DE^d^
Hemoglobin (g/L)^a, e^										
*N* (9 and 18 months)	80	73	76	68		297	87			
Hb at 9 months	89 ± 15	87 ± 14	88 ± 16	89 ± 15	0.925	88 ± 15	88 ± 16	0.805	0.02	1.3
Hb at 18 months	97 ± 17	96 ± 15	98 ± 16	98 ± 15	0.731	97 ± 16	90 ± 15	0.003	0.04	1.5
Plasma ferritin (μg/L)^f^										
*N* (9 and 18 months)	84	75	79	73		311	93			
Unadjusted pF at 9 months	35.9 (29.6, 43.4)	29.7 (24.9, 35.4)	34.1 (28.5, 40.9)	33.0 (26.5, 41.1)	0.127	33.2 (30.2, 36.5)	27.4 (23.3, 32.3)	0.096	0.00	1.0
Adjusted pF at 9 months	23.1 (19.4, 27.4)	18.5 (15.7, 21.8)	21.6 (18.2, 25.5)	19.7 (16.3, 23.8)	0.166	20.7 (19.0, 22.6)	17.0 (14.6, 19.8)	0.038	0.00	1.0
Unadjusted pF at 18 months	44.6 (36.3, 54.7)	47.7 (38.9, 58.5)	34.0 (28.7, 40.2)	42.0 (34.0, 52.1)	0.023	41.7 (37.8, 46.0)	27.0 (22.3, 32.8)	<0.0001	0.04	1.5
Adjusted pF at 18 months	29.5 (24.9, 35.0)^ab^	30.5 (25.2, 36.8)^a^	22.1 (18.5, 26.4)^b^	28.6 (23.3, 35.0)^ab^	0.016	27.4 (25.0, 30.0)	16.9 (14.3, 19.9)	<0.0001	0.06	1.8
Soluble transferrin receptor (mg/L)^g^										
*N* (9 and 18 months)	84	75	79	73		311	93			
Unadjusted sTfR at 9 months	17.8 (15.8, 20.0)	19.7 (17.7, 22.0)	18.4 (16.5, 20.4)	18.4 (14.7, 18.6)	0.286	18.1 (17.1, 19.1)	16.6 (14.8, 18.7)	0.385	0.03	1.4
Adjusted sTfR at 9 months	15.7 (14.0, 17.6)	17.4 (15.7, 19.4)	16.2 (14.6, 18.0)	14.4 (12.9, 16.1)	0.162	15.9 (15.1, 16.8)	14.6 (13.0, 16.3)	0.270	0.03	1.4
Unadjusted sTfR at 18 months	8.8 (8.1, 9.6)	9.1 (8.3, 9.9)	9.0 (8.3, 9.8)	9.2 (8.4, 10.0)	0.860	9.0 (8.6, 9.4)	11.9 (10.9, 13.0)	<0.0001	0.01	1.1
Adjusted sTfR at 18 months	7.8 (7.2, 8.5)	8.0 (7.3, 8.7)	8.0 (7.3, 8.6)	8.2 (7.5, 8.9)	0.828	8.0 (7.7, 8.3)	10.6 (9.7, 11.5)	<0.0001	0.00	1.0
Body iron stores (mg/kg)										
*N* (9 and 18 months)	84	75	79	73		311	93			
Unadjusted BIS at 9 months	1.06 (0.23, 1.88)	0.00 (–0.75, 0.75)	0.76 (0.06, 1.47)	1.02 (0.18, 1.86)	0.069	0.72 (0.33, 1.10)	0.34 (–0.37, 1.05)	0.521	0.01	1.1
Adjusted BIS at 9 months	–0.08 (–0.88, 0.72)	–1.25 (–1.98,–0.52)	–0.44 (–1.13, 0.25)	–0.35 (–1.14, 0.44)	0.120	–0.52 (–0.89,–0.14)	–0.91 (–1.60,–0.22)	0.401	0.01	1.1
Unadjusted BIS at 18 months	4.37 (3.64, 5.10)	4.51 (3.69, 5.34)	3.30 (2.57, 4.03)	4.02 (3.19, 4.84)	0.025	4.05 (3.67, 4.43)	1.48 (0.76, 2.19)	<0.0001	0.05	1.7
Adjusted BIS at 18 months	3.31 (2.66, 3.97)^ab^	3.35 (2.56, 4.14)^a^	2.21 (1.44, 2.98)^b^	3.04 (2.25, 3.83)^ab^	0.019	2.98 (2.61, 3.35)	0.22 (–0.44, 0.88)	<0.0001	0.06	1.8
Zinc protoporphyrin (μmol/mol heme)^h^										
*N* (9 and 18 months)	81	73	79	73		306	92			
Unadjusted ZPP at 9 months	212 (184, 244)	235 (206, 269)	208 (186, 233)	202 (177, 230)	0.703	214 (200, 228)	198 (169, 233)	0.646	0.10	2.2
Adjusted ZPP at 9 months	193 (167, 223)	212 (185, 242)	188 (169, 210)	180 (158, 204)	0.574	193 (181, 206)	180 (154, 210)	0.625	0.09	2.1
Unadjusted ZPP at 18 months	144 (129, 161)	151 (137, 168)	151 (132, 159)	145 (132, 156)	0.920	146 (139, 153)	203 (184, 225)	<0.0001	0.05	1.7
Adjusted ZPP at 18 months	132 (118, 148)	137 (124, 151)	132 (122, 144)	133 (121, 145)	0.946	133 (127, 140)	182 (164, 203)	<0.0001	0.05	1.7

*Hb* hemoglobin concentration, *pF* plasma ferritin, *sTfR* soluble transferrin receptor, *ZPP* zinc protoporphyrin

^a^Adjusted means ± standard deviation, and geometric mean (95% confidence interval); all such values

^b^Values in a row with superscripts with different letters differ *P* < 0.05 using Proc MIXED. Values are adjusted for the random effect of the village

^c^Inter-cluster coefficient calculated based on the cluster (village) variance and the residual variance from proc MIXED

^d^Design effect calculated based on the standard error of the intervention with or without the random effect of the cluster

^e^Hb values adjusted for AGP, CRP, and at 18 months, adjusted additionally for baseline value and study season

^f^pF values adjusted for AGP, CRP, and at 18 months, adjusted additionally for baseline value, baseline iron supplementation, RDT, maternal BMI and study season

^g^sTfR values adjusted for AGP, CRP, age and sex; and at 18 months, adjusted additionally for baseline value, baseline iron supplementation, study season, and minimum meal frequency

^h^ZPP values adjusted for AGP, CRP and sex; and at 18 months, adjusted additionally for baseline value, baseline iron supplementation, maternal BMI, study season, and animal food source

**Table 4:** Effect of SQ-LNS containing different amounts and sources of zinc along with malaria and diarrhea treatment on prevalence of anemia, iron deficiency and iron deficiency anemia in children aged 9 – 18 months

	LNS-Zn0	LNS-Zn5	LNS-Zn10	LNS-TabZn5	*P*-value among 4 intervention groups^b^	IC	NIC	*P*-value between cohorts^b^
% Anemia (Hb <110 g/L)^a^								
At 9 months, *N* (%)	76 (95.0)	69 (94.5)	72 (94.7)	62 (91.2)	0.794	279 (93.9)	80 (91.9)	0.515
At 18 months, *N* (%)	62 (77.5)	57 (78.1)	52 (68.4)	48 (70.6)	0.503	219 (73.7)	80 (91.9)	0.0007
% Low plasma ferritin (pF <12 μg/L)								
Low unadjusted pF at 9 months, *N* (%)	12 (14.3)	10 (13.3)	9 (11.4)	10 (13.7)	0.950	41 (13.2)	11 (11.8)	0.575
Low adjusted pF at 9 months, *N* (%)	18 (21.4)	25 (33.3)	12 (15.2)	17 (23.3)	0.049	72 (23.1)	29 (31.2)	0.126
Low unadjusted pF at 18 months, *N* (%)	4 (4.8)	2 (2.7)	6 (7.6)	6 (8.2)	0.476	18 (5.8)	19 (20.4)	<0.0001
Low adjusted pF at 18 months, *N* (%)	9 (10.7)	6 (8.0)	16 (20.2)	11 (15.1)	0.109	42 (13.5)	30 (32.3)	0.0001
% Elevated soluble transferrin receptor (sTfR >8.3 mg/L)								
High unadjusted sTfR at 9 months, *N* (%)	77 (91.7)	73 (97.3)	75 (94.9)	70 (95.9)	0.355	295 (94.8)	84 (90.3)	0.100
High adjusted sTfR at 9 months, *N* (%)	71 (84.5)	71 (94.7)	73 (92.4)	69 (94.5)	0.080	284 (91.3)	79 (84.9)	0.163
High unadjusted sTfR at 18 months, *N* (%)	38 (45.2)	37 (49.3)	41 (51.9)	36 (49.3)	0.884	152 (48.9)	70 (75.3)	0.001
High adjusted sTfR at 18 months, *N* (%)	31 (36.9)	30 (40.0)	35 (44.3)	31 (42.5)	0.725	127 (40.8)	66 (71.0)	<0.0001
% Low body iron stores (BIS <0 mg/kg)								
Low unadjusted BIS at 9 months, *N* (%)	31 (36.9)	37 (49.3)	33 (41.8)	29 (39.7)	0.260	130 (41.8)	38 (40.9)	0.782
Low adjusted BIS at 9 months, *N* (%)	37 (44.0)^b^	50 (66.7)^a^	44 (55.7)^ab^	39 (53.4)^ab^	0.049	170 (54.7)	58 (62.4)	0.248
Low unadjusted BIS at 18 months, *N* (%)	8 (9.5)	7 (9.3)	11 (13.9)	11 (15.1)	0.641	37 (11.9)	31 (33.3)	0.0001
Low adjusted BIS at 18 months, *N* (%)	9 (10.7)	15 (20.0)	16 (20.3)	16 (21.9)	0.385	56 (18.0)	43 (46.2)	<0.0001
% Elevated capillary zinc protoporphyrin (ZPP >70 μmol/mol heme)								
High unadjusted ZPP at 9 months, *N* (%)	78 (96.3)	71 (97.3)	79 (100.0)	71 (97.3)	1.000	299 (97.7)	84 (91.3)	0.968
High adjusted ZPP at 9 months, *N* (%)	78 (96.3)	71 (97.3)	79 (100.0)	71 (97.3)	1.000	299 (97.7)	84 (91.3)	0.968
High unadjusted ZPP at 18 months, *N* (%)	80 (98.8)	71 (97.3)	78 (98.7)	72 (98.6)	0.844	301 (98.4)	92 (100.0)	0.982
High adjusted ZPP at 18 months, *N* (%)	80 (98.8)	71 (97.3)	78 (98.7)	72 (98.6)	0.844	301 (98.4)	92 (100.0)	0.982
Iron deficiency anemia								
% Low plasma ferritin and anemia								
Low unadjusted pF and anemia at 9 months, *N* (%)	11 (13.8)	10 (13.7)	8 (10.5)	10 (14.7)	0.933	39 (13.1)	10 (11.5)	0.632
Low adjusted pF and anemia at 9 months, *N* (%)	15 (18.8)	24 (32.9)	11 (14.5)	17 (25.0)	0.064	67 (22.6)	27 (31.0)	0.130
Low unadjusted pF and anemia at 18 months, *N* (%)	3 (3.8)	1 (1.4)	5 (6.6)	4 (5.9)	0.449	13 (4.4)	17 (19.5)	<0.0001
Low adjusted pF and anemia at 18 months, *N* (%)	8 (10.0)	3 (4.1)	13 (17.1)	8 (11.8)	0.067	32 (10.8)	25 (28.7)	0.0002
% Elevated soluble transferrin receptor and anemia								
High unadjusted sTfR and anemia at 9 months, *N* (%)	71 (88.8)	68 (93.2)	68 (89.5)	60 (88.2)	0.656	267 (89.9)	75 (86.2)	0.667
High adjusted sTfR and anemia at 9 months, *N* (%)	67 (83.8)	66 (90.4)	66 (86.8)	59 (86.8)	0.427	258 (86.9)	70 (80.5)	0.490
High unadjusted sTfR and anemia at 18 months, *N* (%)	32 (40.0)	29 (39.7)	28 (36.8)	27 (39.7)	0.852	116 (39.1)	63 (72.4)	0.001
High adjusted sTfR and anemia at 18 months, N (%)	26 (32.5)	24 (32.9)	22 (28.9)	24 (35.3)	0.882	96 (32.3)	59 (67.8)	<0.0001

*Hb* hemoglobin concentration, *pF* plasma ferritin, *sTfR* soluble transferrin receptor, *ZPP* zinc protoporphyrin

^a^ *n* (%)

^b^Values in a row with superscripts with different letters differ *P* < 0.05, using Proc GLIMMIX. Values are adjusted for the cluster effect of the village

The presence of inflammation and study season were significantly associated with final Hb concentration, but only the study season modified the effect of intervention group and cohort on final Hb. In particular, Hb concentration increased significantly in IC children who spent ≥5 months in the study during the rainy season (96 g/L in the IC vs. 82 g/L in NIC, *p* = 0.0003), while the Hb response did not differ significantly in children who participated in the study mostly during the dry season (99 g/L in IC vs. 95 g/L in NIC, *p* = 0.86).

### Iron status

Iron status indicators were affected by the presence of inflammation (Tables 3 and 4), so the results are presented after adjusting for these effects. The estimated prevalence of iron deficiency differed by iron status indicator. In particular, at 9 months, 25% of the children had low adjusted pF, 90% had high adjusted sTfR, 56% had low adjusted BIS and 96% had high adjusted ZPP. 24% of children had IDA as defined by the simultaneous presence of anemia and low adjusted pF, and 85% had IDA as defined by anemia and elevated adjusted sTfR.

There was no effect of supplementing different amounts and sources of zinc on any of the iron status indicators except for pF and BIS. Children who received LNS-Zn10 had a lower adjusted geometric mean pF and BIS compared to LNS-Zn5 (22.1 μg/L vs. 30.5 μg/L, *p* = 0.034 for pF; and 2.21 mg/kg vs. 3.35 mg/kg, *p* = 0.047 for BIS), although the prevalence of low adjusted pF and low adjusted BIS was not significantly different among intervention groups. In a 5-group analysis, adjusted geometric mean pF of children who received LNS-Zn10 was not significantly different from children in the NIC (*p* = 0.298).

**Fig. 2:**
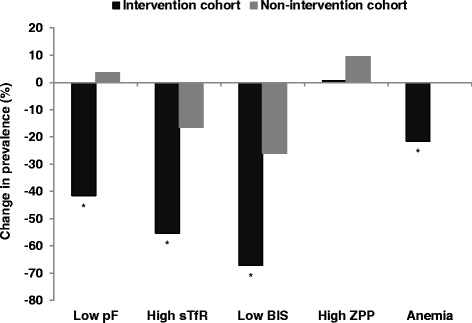
Effect of SQ-LNS combined with malaria and diarrhea treatment on % relative change in prevalence of adjusted indicators of iron deficiency and anemia from 9 to 18 month of age in rural Burkinabe children. * Significantly different between the two cohorts (p < 0.05)

The prevalence of ID at 18 months, as defined by adjusted pF, was significantly different between the two cohorts (*p* < 0.0001). In particular, the prevalence of ID fell from 23% to 14% in the IC (a relative change of −42%), whereas it did not change in the NIC (Fig. 2). Similarly, the prevalence of ID, when defined by adjusted BIS, was significantly different between the two cohorts at 18 months and only fell significantly in the IC over the course of the study (Fig. 2). In contrast, the prevalence of elevated adjusted ZPP did not differ by cohort and increased from 96% to 99% (*p* = 0.01). The difference in prevalence of ID between IC and NIC was similar whether or not the indicators were adjusted for inflammation (Table 4). IDA was lower in both cohorts at 18 months, but decreased significantly only in the IC (IDA defined by pF decreased relatively by 51% in the IC vs. 7% in NIC, and IDA defined by sTfR decreased relatively by 63% in the IC vs. 16% in the NIC).

### Vitamin A status

At baseline, adjusted RBP concentration did not differ by intervention group or cohort, with a geometric mean (95% confidence interval) of 0.97 (0.94, 1.00) μmol RBP/L. A total of 47% of the children had low adjusted plasma RBP concentrations at 9 months. Adjusted RBP concentration decreased more from 9 to 18 months in the NIC than in the IC (a relative change of −9 vs −4%, *p* = 0.014). Although the mean concentration at 18 months of, and the change in adjusted RBP was significantly different between the cohorts, the final prevalence of low RBP did not differ significantly (Table 5). Mean adjusted RBP at 18 months was not significantly different by intervention group (*p* = 0.056). RBP concentration at 18 months was significantly related to baseline RBP concentration, inflammation indicators, maternal education, and more frequent reported breastfeeding at 9 months, but none of these variables significantly modified the effects of intervention group or cohort.

**Table 5:** Effect of SQ-LNS containing different amounts and sources of zinc provided along with malaria and diarrhea treatment on retinol binding protein concentrations and prevalence of low retinol binding protein concentrations in children at 9 and 18 months, by study group

	LNS-Zn0	LNS-Zn5	LNS-Zn10	LNS-TabZn5	*P*-value among 4 intervention groups^a, b^	IC	NIC	*P*-value between cohorts^a, b^	ICC^c^	DE^d^
Retinol binding protein (μmol/L)^e^										
*N* (9 and 18 mo)	84	75	79	73		311	93			
Unadjusted RBP at 9 months	0.98 (0.91, 1.05)	0.86 (0.79, 0.94)	0.94 (0.88, 1.01)	0.87 (0.81, 0.94)	0.197	0.92 (0.88, 0.95)	0.88 (0.82, 0.94)	0.618	0.05	1.7
Adjusted RBP at 9 months	1.04 (0.97, 1.12)	0.93 (0.85, 1.01)	1.01 (0.95, 1.08)	0.95 (0.88, 1.02)	0.171	0.98 (0.95, 1.02)	0.94 (0.88, 1.00)	0.555	0.05	1.7
Unadjusted RBP at 18 months	0.86 (0.81, 0.92)	0.86 (0.80, 0.92)	0.92 (0.86, 0.98)	0.95 (0.90, 1.00)	0.044	0.89 (0.87, 0.92)	0.79 (0.74, 0.84)	0.004	0.03	1.4
Adjusted RBP at 18 months	0.91 (0.86, 0.96)	0.92 (0.86, 0.98)	0.97 (0.91, 1.03)	0.99 (0.94, 1.05)	0.056	0.94 (0.92, 0.97)	0.86 (0.81, 0.91)	0.015	0.01	1.1
% Low retinol binding protein (RBP <0.94 μmol/L)^e^										
*N* (9 and 18 months)	84	75	79	73		311	93			
Low unadjusted RBP at 9 months, *N* (%)	41 (48.8)	44 (58.7)	42 (53.2)	48 (65.7)	0.456	175 (56.3)	53 (57.0)	0.892	–	–
Low adjusted RBP at 9 months, *N* (%)	32 (38.1)	40 (53.3)	32 (40.5)	38 (52.0)	0.240	142 (45.7)	48 (51.6)	0.509	–	–
Low unadjusted RBP at 18 months, *N* (%)	54 (64.3)	51 (68.0)	45 (57.0)	34 (46.6)	0.028	184 (59.2)	66 (71.0)	0.052	–	–
Low adjusted RBP at 18 months, *N* (%)	46 (54.8)	41 (54.7)	36 (45.6)	31 (42.5)	0.280	154 (49.5)	53 (57.0)	0.332	–	–

RBP, retinol binding protein

^a^Geometric mean (95% confidence interval), n (%). Values in the same row with different superscript letters are significantly different (*P* < 0.05)

^b^Adjusting for the random effect of the village and baseline value

^c^Inter-cluster coefficient calculated based on the cluster (village) variance and the residual variance from proc MIXED

^d^Design effect calculated based on the standard error of the intervention with or without the random effect of the cluster

^e^RBP adjusted for CRP and AGP concentrations; and at 18 months for baseline value, breastfeeding at baseline and women education

At 18 months, 22% of children reportedly consumed vitamin A-rich fruits at least 3 days during the previous week, and none reportedly consumed vitamin A-rich vegetables with this frequency. There was no relationship between reported consumption of vitamin A rich fruits and RBP concentration. Twelve percent of children in the IC reportedly received a high-dose vitamin A supplement during the month preceding the biochemistry visit at 18 months. However, reported receipt of high-dose vitamin A supplement was not associated with RBP concentration at 18 months. Similarly, RBP concentration at 18 months was not significantly related to baseline pZC, and pZC did not modify the effect of intervention group or cohort on final RBP concentration.

## Discussion

Different amounts of zinc provided either in SQ-LNS or as a dispersible zinc tablet did not affect the final pZC or other markers of micronutrient status, except for the lower final mean pF concentration and lower BIS in children who received LNS-Zn10 compared to those who received LNS-Zn5. By contrast, SQ-LNS, provided along with malaria and diarrhea treatment in the intervention cohort, increased the children’s final iron status, Hb concentration and vitamin A status, compared with those in the non-intervention cohort. The intervention package also reduced the prevalence of iron deficiency, anemia and IDA, compared with the NIC, although most children remained anemic at 18 months. Hb concentration increased significantly more among children in the IC who participated in the study mostly during the rainy season, when food is generally less available and malaria and diarrhea are more common, suggesting that the benefit may be greatest during periods of food insecurity and/or more frequent infections.

One third of children had subclinical zinc deficiency at baseline and more than half of all children had subclinical zinc deficiency at endline. Thus, zinc deficiency is a public health concern in the study population [40]. Nevertheless, the lack of a change in pZC after zinc supplementation with the dispersible tablet differs from the results of multiple other studies [10], including a short-term study completed in the same geographic region in which supplements were given under direct observation and produced a sizeable increase in pZC [28]. The lack of change in pZC among children supplemented with zinc tablets in the present study could indicate low adherence to supplementation with tablets or failure to provide the tablets between meals. In fact, we have previously reported that tablet adherence was less than 30% in a subgroup of children who were observed for 12 h at home; and more than half of the time, the tablet was given less than 30 min after either a meal or SQ-LNS had been served, contrary to what was recommended according to the study protocol [24]. The lack of change in pZC among children who received additional zinc through SQ-LNS is consistent with results from most previous trials that provided supplemental zinc mixed with food [19, 41–43]. Zinc provided with food is presumably less well absorbed than when provided as a tablet or syrup apart from meals, or the absorbed zinc is metabolized differently post-absorption.

Zinc supplementation had no effect on Hb concentration and anemia prevalence, regardless of the dose or form of supplementation. This is in agreement with a meta-analysis of zinc supplementation trials, which found that daily zinc supplementation (10–20 mg) among children <15 years of age had no effect on Hb concentration [44]. Zinc supplementation also had no effect on most iron status indicators except for the lower pF concentration and lower BIS found in children who received LNS-Zn10, possibly due to inhibition of iron absorption in the presence of higher amounts of zinc. Nevertheless, iron status was increased by concurrent supplementation of zinc and iron despite the slightly smaller magnitude of response with the higher dose of zinc [10, 11].

Provision of supplemental zinc did not significantly affect the final prevalence of vitamin A deficiency or mean adjusted RBP at 18 months. Previous studies on the impact of adding zinc to vitamin A supplements on vitamin A status have yielded inconsistent results [12–14]. Smith suggested that the effect of zinc on response to vitamin A supplementation may be conditioned by the severity of zinc deficiency [45]. This was not confirmed in our study. Although zinc deficiency was a public health concern in the study population baseline pZC had no effect on the response of RBP to zinc supplementation.

Anemia prevalence was reduced by 20% in the IC, but most children were still anemic at 18 months, and anemia remained a severe public health problem in this population. Because SQ-LNS provided the recommended daily allowance of more than 20 micronutrients [15], including iron, vitamin A and vitamin B_12_, other factors, such as malaria, intestinal helminthes, other infections and hemoglobinopathies may have contributed to the high anemia burden [46]. However, in a separate analysis including only children from the IC, the frequency of malaria episodes during the 9 months of the intervention was not associated with the change in Hb concentration or anemia prevalence at 18 months [47].

At 18 months, the intervention reduced the prevalence of ID by 39–45% compared with the NIC, depending on the ID indicator. Almost all children had high ZPP at 18 months, which suggests that ZPP may not be a useful indicator of iron status in this population due to the high burden of malaria and other infections. Previous studies indicate that ZPP values may be falsely increased in individuals with infections, chronic inflammation, hemoglobinopathies and high lead intakes [48–52].

Our findings regarding the effects of SQ-LNS on Hb and iron status are similar to results from LNS interventions in other low-income countries. In a study in Ghana, 6 months old infants supplemented for 6 months with SQ-LNS containing 9 mg iron/daily dose also had increased iron status and lower anemia prevalence compared to a non-intervention group [19]. In a randomized clinical trial in Malawi, moderately malnourished children 6–18 months of age who received 25 g/d of a milk-based fortified spread for 12 weeks had a greater change in Hb (mean ± SD: 11 ± 21 g/L) compared to children who did not receive any supplement (1 ± 20 g/L) [53]. In contrast, supplementation of 6–18 months old children with MQ-LNS (46 to 70 g, containing 400 μg vitamin A, 9 mg iron and 9 mg zinc per 46 g) did not affect iron status, Hb concentration or prevalence of anemia during an efficacy trial in rural Honduras compared to a control population who received, similarly to the intervention population, food vouchers and nutrition counselling [20]. Our findings are also similar to results of trials of micronutrient powders (MNP), which provide 12 mg iron, 300 μg retinol and 5 mg zinc per daily dose. A recent meta-analysis found that daily MNP supplementation among 6–23 months old children reduced anemia prevalence by 31% and iron deficiency by 51% compared to placebo or no intervention [54, 55].

The intervention package increased mean plasma RBP concentration, but did not affect the prevalence of low RBP concentration. Similarly, supplementing 6–18 months old Honduran children with MQ-LNS containing 400 μg vitamin A significantly increased plasma retinol concentration after 6 months compared to a control group, although this difference was no longer present after 12 months of supplementation [20].

Our study has several strengths including: 1) the different micronutrient status indicators examined; 2) the large sample size; 3) the continuous training and supervision of field workers responsible for collecting data and obtaining the biological samples; and 4) the post-hoc calculated design effect (1.0–1.7) was accounted for by the inflated sample size in the NIC, which shows that our effective sample size has not been reduced greatly for most of the indicators. We also recognize several limitations of our study, which include the lack of an illness treatment only cohort to differentiate between the effect of SQ-LNS supplementation and the effect of malaria and diarrhea treatment. In addition, due to the lack of information on malaria infection at 18 months, we could not adjust pF and RBP for the presence of asymptomatic malaria, as recently proposed by Wessells et al. [56].

## Conclusions

Our results indicate that supplemental zinc provided in SQ-LNS or as a dispersible tablet increased Hb and vitamin A status. Although children who received SQ-LNS containing 10 mg zinc had a lower pF response than those who received SQ-LNS containing less zinc, there were no group-wise differences in the final prevalence of ID or anemia in the IC. The lack of a change in pZC after supplementation with a zinc tablet containing 5 mg zinc and with SQ-LNS containing 5 or 10 mg zinc suggests possible low adherence to the dispersible tablet and/or poor absorption of supplemental zinc when provided with food, or different post-absorptive metabolism of zinc when absorbed. Despite the lack of an effect of zinc supplementation in this study population, SQ-LNS along with malaria and diarrhea treatment was beneficial in reducing the prevalence of ID and anemia and increasing vitamin A status in young children.

## Abbreviations

AGP: α-1-acid glycoprotein
BIS: Body iron stores
CRP: C-reactive protein
IC: Intervention cohort
ID: Iron deficiency
IDA: Iron deficiency anemia
NIC: Non-intervention cohort
pF: Plasma ferritin
pZC: Plasma zinc concentration
RBP: Retinol-binding protein
ROH: Retinol
SQ-LNS: Small-quantity lipid-based nutrient supplement
sTfR: Soluble transferrin receptor
ZPP: Zinc protoporphyrin
